# Rapid parasitological indicators as practical biosecurity tools in inland Nile tilapia (*Oreochromis niloticus*) aquaculture: A national multiregional two-season survey in Saudi Arabia

**DOI:** 10.14202/vetworld.2025.4056-4068

**Published:** 2025-12-23

**Authors:** Mohammad Nafi Solaiman Al- Sabi, Heba Ibrahim Abdel-mawla, Jamal Hussen, Ibrahim Fahad Albokhadaim, Kurt Buchmann

**Affiliations:** 1 Department of Basic Veterinary Medical Sciences, Faculty of Veterinary Medicine, Jordan University of Science and Technology, P. O. Box 3030, Irbid 22110, Jordan; 2 Department of Fish Diseases, Animal Health Research Institute, Agricultural Research Center, Cairo, Egypt; 3 Department of Microbiology, College of Veterinary Medicine, King Faisal University, Al-Ahsa, Saudi Arabia; 4 Department of Biomedical Sciences, College of Veterinary Medicine, King Faisal University, Al-Ahsa, Saudi Arabia; 5 Department of Veterinary and Animal Sciences, Faculty of Health and Medical Sciences, University of Copenhagen, Frederiksberg C, Denmark

**Keywords:** aquaculture biosecurity, inland aquaculture, Nile tilapia, parasitological screening, parasite prevalence, Saudi Arabia, seasonal variation, water quality indicators

## Abstract

**Background and Aim:**

The inland aquaculture sector in Saudi Arabia has expanded rapidly, with Nile tilapia (*Oreochromis niloticus*) becoming the dominant cultured species. However, limited national surveillance has restricted early detection of parasitic infestations that could indicate breaches in farm-level biosecurity. This study aimed to evaluate low-cost parasitological screening as a practical indicator of biosecurity performance by assessing parasite prevalence, diversity, and predictors across inland farms during two seasons.

**Materials and Methods:**

A sample of 30 fish from each farm was examined from 25 inland aquaculture sites sampled in summer and winter (2022–2023). External and internal parasitic infestations were evaluated through wet smears of the skin, fins, and gills, and by compression techniques for digeneans. Water quality parameters, serum cortisol levels, and farm management data were recorded. Parasites were identified to the genus-level. Predictors of infestation were analyzed using binary logistic regression followed by generalized linear mixed models.

**Results:**

Six parasite groups were detected, with infestations recorded on all farms. *Trichodina* spp. showed the highest farm-level prevalence (96%) and fish-level prevalence (54.3%), followed by *Cichlidogyrus* spp. (92% of farms; 56.9% of fish). *Dactylogyrus*, *Ambiphyra*, *Gyrodactylus*, and *Centrocestus* were variably present, with the eastern region displaying the lowest diversity. Most infestations were mild. Significant predictors varied by parasite type: summer season increased the likelihood of *Centrocestus*, *Dactylogyrus*, *Cichlidogyrus*, and *Ambiphyra*; low pH strongly predicted monogenean and sessile ciliate infestations; dissolved oxygen and fish length also contributed to parasite-specific patterns. No clear association existed between infestation and elevated cortisol levels.

**Conclusion:**

This nationwide two-season assessment shows that simple parasitological screening, especially for *Trichodina* and *Cichlidogyrus*, offers quick, affordable signs of biosecurity breaches in inland *O. niloticus* aquaculture. The presence of parasites indicates past or current risks of pathogen introduction, highlighting the importance of better quarantine procedures, water quality management, and following national biosecurity guidelines. Regular quarterly parasitological checks, combined with water quality testing and improved farm hygiene, can enhance early detection and reduce the spread of disease across Saudi Arabia’s aquaculture industry.

## INTRODUCTION

Global fish production continues to rise, mainly driven by aquaculture activities, as capture fisheries have plateaued in recent decades [1]. Meanwhile, global demand for fish has grown, a trend also seen at the local level in several Middle Eastern countries, where consumption reports highlight a strong need to expand fish production [2]. This increasing demand calls for the development and implementation of comprehensive aquaculture strategies in the region. Currently, aquaculture production in the Middle East remains the lowest worldwide in volume, prompting authorities to promote greater investment in the sector [3].

Nile tilapia (*Oreochromis niloticus*) is a suitable species for freshwater farming in the region. The first hatchery was established at a research center in Riyadh in 1980, and fingerlings were later distributed to local farms in 1983 [3]. The number of farms grew from three in 1983 to more than 80 in 2021, and further expansion is expected because over 200 license applications are currently being processed by the Saudi Ministry of Environment, Water, and Agriculture (MEWA) [1, 4]. *O. niloticus* makes up 98% of the cultured fish species in the country, with 84 inland farms actively producing fish [5]. Although tilapia can be imported from approved international suppliers [6], current national production mainly depends on locally established broodstock. As production increases, focus must be placed on disease prevention and strategies to reduce the risk of pathogen introduction.

The use of fish parasites as bioindicators is widely employed to differentiate marine fish stocks [7, 8]. Similarly, basic fish parasitological examinations, identifying parasites to the genus-level, can reveal potential breaches in farm biosecurity measures and suggest the risk of infestation with additional pathogens (e.g., unspecified bacteria and viruses) associated with introduced fish. Although the presence of parasitic infections does not necessarily correlate with viral and/or bacterial infections, parasite burdens may predispose fish to secondary bacterial, fungal, and viral diseases by stimulating immune responses that increase susceptibility to other microbial agents and potentially reduce the effectiveness of vaccination programs [9].

Previous studies have indicated that tilapia diseases can be caused by various viruses and bacteria [10] and by over 430 parasitic pathogens [11], with 27 species documented in the Middle East and nearby regions [12]. Several reports have detailed parasitic infections in inland tilapia aquaculture in Saudi Arabia [13–17].

Although several regional studies have reported ciliates, monogeneans, and digenean parasites infesting farmed *O*. *niloticus* in Saudi Arabia, these investigations remain geographically limited and lack seasonal or nationwide representation. No previous study has conducted a comprehensive, multi-regional, multi-seasonal survey integrating parasite prevalence, infestation intensity, and species diversity across inland aquaculture. This absence of baseline epidemiological data restricts the understanding of how parasitic infestations spread across farms, how they relate to management practices and environmental conditions, and how they may indicate weaknesses in biosecurity. Although full diagnostic investigations for viral and bacterial pathogens require advanced laboratory infrastructure, such resources are not uniformly available across all farms. As highlighted by Buchmann [18], simple parasitological examinations, identifying parasites with basic microscopy, can serve as low-cost, practical indicators of previous exposure to pathogenic risks, thereby revealing potential breaches in biosecurity. However, this approach has not yet been systematically evaluated at the national level. Consequently, a critical research gap exists regarding the use of low-technology parasitological screening as a tool to assess biosecurity performance, identify potential pathways of pathogen introduction, and support national surveillance frameworks in Saudi Arabian inland tilapia aquaculture.

This study aimed to produce the first nationwide, multi-seasonal, and multi-regional assessment of parasitic infestations in inland *O. niloticus* aquaculture in Saudi Arabia using inexpensive parasitological methods. Specifically, the study sought to: (i) determine the prevalence, diversity, and infestation severity of major parasitic groups (ciliates, monogeneans, and digeneans) across farms located in the central, eastern, and western regions; (ii) examine the relationship between parasite occurrence and key abiotic factors, including water temperature, pH, dissolved oxygen, and nitrogen compounds; (iii) investigate the connection between parasite infestation and physiological stress markers, such as serum cortisol levels; and (iv) explore the links between parasitic patterns, farm management practices, water-use strategies, and potential pathways for parasite introduction and spread. By combining parasitological, environmental, and management data, the study aimed to assess whether parasites, especially common genera like *Trichodina* and *Cichlidogyrus*, could serve as practical, rapid, and affordable sentinel indicators of biosecurity in inland aquaculture. The main goal was to develop evidence-based recommendations to support national biosecurity policies and guide the future implementation of standardized routine screening methods across Saudi Arabian tilapia farms.

## MATERIALS AND METHODS

### Ethical approval

All animal procedures were conducted in accordance with the 2010/63/EU Directive on the protection of animals used for scientific purposes [19] and the ethical principles of the Kingdom of Saudi Arabia (KSA). The Institutional Animal Care and Use Committee of King Faisal University approved the study under approval number KFU-REC-2024-SEP-ETHICS2477. Fish were handled by trained personnel to minimize stress and discomfort. Euthanasia was performed using a sharp percussive blow to the head, followed immediately by blood collection. All sampling and handling procedures adhered to the Animal Research: Reporting of *In Vivo* Experiments 2.0 guidelines, the World Organization for Animal Health (WOAH, previously known as OIE) Aquatic Animal Health Code (2021), and the National Fish Biosecurity Manual [6]. Live fish were not retained for experimental infection or prolonged holding following sampling.

### Study period and location

The study was conducted from May 2022 to June 2023. This study combined farm management practices, water chemistry, fish stress physiology, and genus-level parasitology within a multi-layered analytical framework. It used a prospective cohort design with two-season sampling (summer and winter) at the farm-level from summer 2022 to summer 2023 in the KSA. Inland aquaculture farms in the KSA are mainly located in the central, eastern, and western regions, with limited presence in the northern and southern areas.

**Figure 1:**
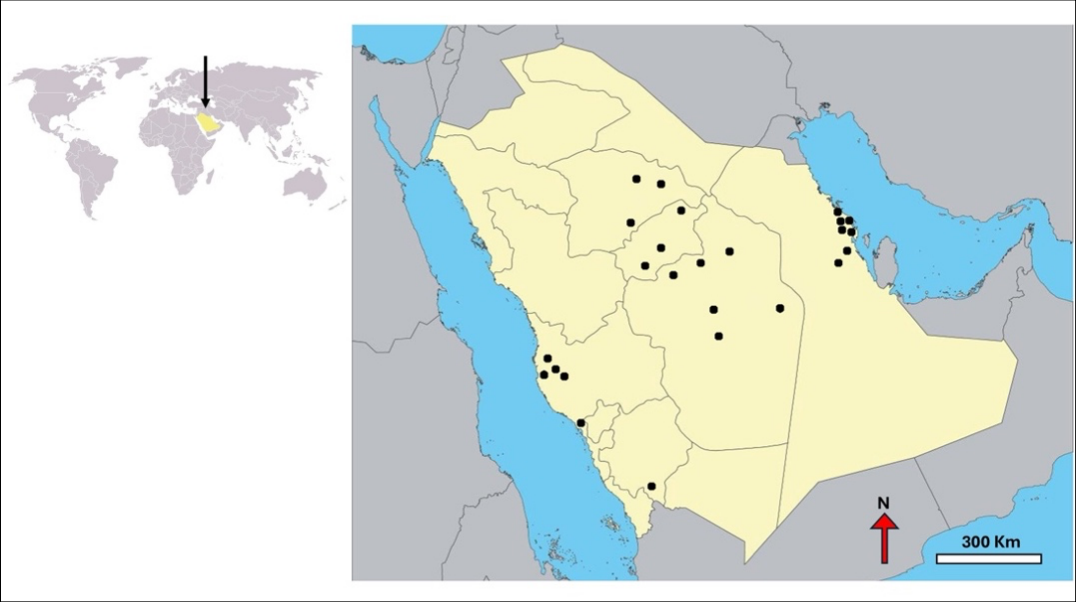
A map of Saudi Arabia showing the locations of the sampled 25 inland tilapia farms from 2022 to 2023 [The map was generated using QGIS software].

A total of 25 farms were chosen from the 84 actively producing farms, representing 29.8% of all farms, based on geographic distribution (central, eastern, and western regions; Figure 1), production type (hatcheries and grow-out farms), capacity, presence of mixed-species systems, willingness to participate, and recommendations from the MEWA. Priority was given to key hatcheries (n = 15) that supply fingerlings to smaller producers (n = 10).

Farms in the eastern region (n = 7) are situated in low-elevation desert areas rich in groundwater and near the Arabian Gulf. Farms in the central region (n = 12) are located on the elevated Najd plateau, an arid zone dependent on deep-water sources. Farms in the Western Region (n = 6) are positioned in mountainous areas near the Red Sea.

### Sampling

Sample size determination followed the OIE guidelines and the Animal Health Law 2016/429, including its implementation under Regulation (EU) 2016/429 and Commission Delegated Regulation (EU) 2020/689 for Category C diseases. Assuming a medium risk of contracting a serious disease, epidemiological algorithms recommended sampling 30 fish per farm per visit per season, with a maximum of 10 fish per pooled sample.

The sampling team included four trained postgraduate students (PhD/MSc). Before field sampling, experts from the OIE held formal training sessions on fish handling and sampling, then provided hands-on supervision during the first farm visit.

### Fish selection, randomization, and blinding

Fish were selected randomly, giving priority to moribund individuals showing clinical signs when available. Raceways (alternatively called ponds, tanks, production units, or basins) with previous mortality reports were sampled systematically using netting at 1-meter intervals. Fish of various ages (2–35 cm) were included.

For blinding, each fish received a barcode label and was placed in an ice chest for transport to the research laboratory. Each farm was sampled twice, once in summer and once in winter.

### Questionnaire on farm management and biosecurity

Farm-level management information was gathered in Arabic through structured interviews with the farm manager or administrative staff during each visit. Missing responses were clarified by phone. Data were recorded separately for each seasonal visit using a structured questionnaire (Supplement 1). Variables included water source, water treatment practices, fish sourcing, species composition, seasonal mortality rates, disease control measures, and feed management.

### Water sampling and quality assurance

Water parameters were measured on-site during each visit and season using a portable multi-parameter device (IP67 COMBO Model 86031, AZ Instrument Corp., Taichung, Taiwan) to record pH, temperature, dissolved oxygen (DO), transparency, conductivity, and salinity.

Duplicate water samples (500 mL each) were collected from at least two raceways per farm at approximately 1-meter depth in the morning to minimize diurnal DO fluctuations. Ammonia, nitrate/nitrite, and heavy metals were analyzed immediately using a 16-in-1 Drinking Water Test Kit (SJ WAVE, Australia). Sampling was conducted without preservatives. The interpretation of the water quality values for Nile tilapia farms was based on previous literature [20–22].

Instrument calibration was performed before sampling, at the end of the first season, and at the completion of field activities. Additionally, water samples from the first sampled farm were analyzed in a third-party laboratory at King Faisal University for quality assurance.

### Blood collection and cortisol assay

Fish were collected using a hand net and immediately euthanized according to ethical procedures. Blood was drawn within 15 min through caudal vein puncture with a 25-gauge needle into serum vacutainer tubes (Guangzhou Improve Medical Instruments Co., China). Samples were kept on ice (~4°C) and transported to the lab within 8 h or stored temporarily in farm refrigerators.

Serum was separated by centrifugation at 1,000 × *g* for 10 min and stored at –80°C. Cortisol concentrations were measured using a commercial enzyme-linked immunosorbent assay (ELISA) kit (DRG® Cortisol ELISA Kit, DRG, USA). Standard curves ranged from 0 to 80 μg/dL. Assay sensitivity was 0.1 μg/dL (6.0 nmol/L), with intra- and inter-assay variability of 3.2% and 6.5%, respectively.

Cortisol levels in *O. niloticus* were categorized as described by Samaras and Pavlidis [23]:

- Low: <7.6 μg/dL
- Medium: 7.6–10.6 μg/dL
- High: >10.6 μg/dL.

Values below the Limit of Quantification were recorded as 0 μg/dL.

### Parasitological examination

Parasitological assessment focused on the eyes, skin, fins, gills, and muscles. Wet smears from the skin, fins, and gills were used to detect external parasites attached to epithelial cells and mucus. Each fish was smeared onto a slide, immediately fixed in 100% methanol, and transported at ambient temperature.

Slides were stained with Giemsa (1:9 dilution) for 20 min, rinsed, air-dried, and examined under a compound microscope (Optika® B-66, Italy) at ×40 and ×100 oil immersion magnification [24].

Parasite identification was performed by a fish parasitologist with over 20 years of experience, using established diagnostic keys [25], and confirmed by a second expert with more than 35 years of experience. Identification was limited to the genus-level, aligning with the study objectives.

Muscle trematodes were examined by exposing muscle tissue, visually inspecting for discoloration, and preparing compression slides [26]. Gills of frozen fish were examined using a stereomicroscope (Leica EZ4E; 7–40×).

Infestation intensity was graded as follows [13]:

- Mild (+): 1 parasite in up to 10 fields
- Moderate (++): 2–5 parasites in up to 10 fields
- Severe (+++): >5 parasites in up to 10 fields

### Statistical analyses

Data were compiled in Excel® 2021 (Microsoft Corp., Washington, USA) and analyzed using SPSS® v30.0 (IBM Corp, USA). Prevalence and 95% confidence intervals (CIs) were calculated based on binomial distribution assumptions [27].

A two-stage modeling approach was implemented:

- Binary logistic regression to identify significant predictors for each parasite (season, fish length, pH, temperature, DO, nitrate, nitrite, cortisol).
- Generalized linear mixed models (GLMMs) were constructed using significant variables from step 1, incorporating farm ID as a random intercept to account for clustering.

Adjusted odds ratios (OR), 95% CIs, and p-values were calculated. Model fit was assessed using the Akaike Information Criterion (AIC), with lower AIC values indicating a better fit.

Missing data were handled with likelihood-based estimation. Variables with more than 10% missing data were excluded during sensitivity analysis. Only complete cases were used in the final GLMM.

## RESULTS

### Management systems in inland aquaculture

The farm areas ranged from 1,500 m² to 500,000 m², and the designs of the rearing tanks varied across farms. Most farms used concrete raceways of different sizes, while a few used fiberglass raceways (Table 1). The number of raceways ranged from 12 to 277, and monthly fish production ranged from 3 to 32 metric tons. Raceways were either outdoors without shade (11 farms; 44%) or indoors or shaded (14 farms; 56%). Only four farms (16%) operated dedicated quarantine raceways.

**Table 1:** Summary of the management systems in 25 inland tilapia aquaculture sites located in different provinces in the Kingdom of Saudi Arabia from 2022 to 2023.

Item	Variable	Percentage (n)
Culture system	Low intensity	52 (13)
	Intensive	40 (10)
	Hyper-intensive	8 (2)
Type of fish raceways used	Concrete	56 (14)
	Concrete and Sand	28 (7)
	Plastic	8 (2)
	Plastic and concrete	8 (2)
Fish raceway coverage	Shaded outdoor	56 (14)
	Not-shaded outdoor	44 (11)
Stock source of fish	Internal breeding only	68 (17)
	Local breeders inside the KSA	20 (5)
	Internal and local breeders	8 (2)
	Importing from outside the KSA (Thailand)	4 (1)
Mixed fish species	No	68 (17)
	Yes	32 (8)
Aeration	Fountains	8 (2)
	Impellers	4 (1)
	Aeration pumps and fountains	20 (5)
	Aeration pumps and impellers	28 (7)
	Aeration pumps, fountains, and impellers	36 (9)
	Aeration pumps and oxygen pumping in the water	4 (1)
Source of water	Wells	100 (25)
Treatment with water after use	Filtration–recirculating aquaculture system	4 (1)
	No treatment	96 (24)
Reuse of water aquaculture	Reused in aquaculture without treatment	28 (7)
	Pumped out to agricultural activities without treatment	72 (18)
Disinfestation method	Slacked lime	16 (4)
	Formalin	16 (4)
	Potassium permanganate	16 (4)
	Lack of lime and formalin	12 (3)
	Formalin, lime slack, and potassium permanganate	4 (1)
	Washing and scrubbing with detergents and water	36 (9)
Mortality rates	Summer	1–4
	Winter	1–6

Among the growing systems, 13 farms (53%) maintained low stocking densities (<50 fish/m³), 10 farms (40%) used intensive systems (51–100 fish/m³), and two farms (8%) operated under hyper-intensive conditions (>100 fish/m³). Eight farms (32%) cultured multiple species, including red tilapia, carp, ornamental fish, and catfish, in separate raceways. Reported mortality rates ranged from 1%–4% in summer and 1%–6% in winter.

### Construction and management of farms

All sampled *O. niloticus* farms relied solely on groundwater. Although farms did not routinely monitor water quality with digital instruments, water in most raceways was regularly renewed or maintained under continuous-flow systems. In seven farms (28%), untreated effluent water from one raceway was reused in other raceways, increasing the risk of parasite transmission. All farms reused outlet water for agricultural activities.

Only one farm (4%) operated a functioning recirculating aquaculture system (RAS), while four farms (16%) were in the process of establishing one. At the end of each production cycle, raceways were drained, washed, and manually scrubbed with or without detergents. Chemical disinfection was used in 16 farms (64%) with formalin, slaked lime, or potassium permanganate for 3–7 days before the final rinse. The remaining nine farms (36%) relied solely on air-drying raceways for 3–7 days.

### Parameters of water quality

Most raceways showed acceptable pH, ammonia, and hardness levels, while suboptimal temperature, DO, nitrate, and nitrite levels were recorded. Normal temperature values were observed in 54% of raceways, whereas 26% had low temperatures and 20% had high temperatures, ranging from 12.5°C in winter to 30.3°C in summer. Acceptable DO levels were found in 76% of samples, with the lowest recorded DO level at 0.32 mg/L. Nitrate and nitrite concentrations were normal in 60% of raceways; the remaining 40% showed elevated levels (Table 2).

**Table 2:** Water quality parameters recorded during a one-year cohort study of 25 inland tilapia aquaculture in Saudi Arabia from 2022 to 2023.

Variables	Season	Temperature	pH	DO	Ammonia	Nitrates	Nitrites	Hardness
Average normal	Summer	81	98%	84%	100%	76%	76%	100%
	Winter	66%	94%	90%	100%	74%	74%	100%
Average low	Summer	3%	0%	16%	0	NA	NA	0
	Winter	28%	4%	10%	0	NA	NA	0
Average high	Summer	16%	2%	NA	0	24%	24%	0
	Winter	6%	2%	NA	0	26%	26%	0

DO = Dissolved oxygen, NA = Not applicable.

### Blood cortisol levels

Since no linear relationship was observed between cortisol levels and parasite infestation, serum cortisol values were grouped. Overall, 73.3% of fish had normal cortisol levels (<7.6 μg/dL), while 13.6% displayed high levels (>10.6 μg/dL), and 13.1% showed moderate levels (7.6–10.6 μg/dL). Most parasitized fish had cortisol concentrations within the normal range (Table 3) [13, 18], suggesting no direct link between parasite presence and increased cortisol.

**Table 3:** Prevalence rate (95% CI), recorded during a one-year cohort study in 25 inland Nile tilapia aquaculture in Saudi Arabia from 2022 to 2023.

Parasite group	Ciliates	Ciliates	Monogeneans	Monogeneans	Monogeneans	Digeneans
Variables	*Trichodina*	*Ambiphyra*	*Cichlidogyrus*	*Dactylogyrus*	*Gyrodactylus*	*Centrocestus*
Prevalence rate (fish-level)	54.3 (49.6–59)	10.6 (7.7–13.5)	56.9 (54–59.8)	22.6 (20.1–25.1)	0.9 (0.3–1.5)	5.8 (4.4–7.2)
Prevalence (farm-level)	96 (24/25)	36 (9/25)	92 (23/25)	80 (20/25)	12 (3/25)	12 (3/25)
Mild infestation	32.4 (28–36.8)	8.3 (5.7–10.9)	36.9 (34.1–39.8)	10.0 (8.2–11.8)	0.8 (0.2–1.4)	3.3 (2.3–4.4)
Moderate infestation	12.0 (8.9–15.1)	1.8 (0.5–3.1)	13.7 (11.7–15.7)	8.4 (6.8–10.0)	0.1 (0–0.3)	0.9 (0.3–1.5)
Severe infestation	9.9 (7.1–12.7)	0.5 (0.3–0.7)	6.4 (5.0–7.8)	4.3 (3.1–5.5)	0.0	1.5 (0.8–2.2)
Infestations in the summer	16.3 (12.8–19.8)	1.4 (0.3–2.5)	30.8 (28.1–33.5)	9.7 (7.9–11.5)	0.6 (0.1–1.1)	2.3 (1.4–3.2)
Infestations in the winter	37.9 (33.3–42.5)	9.2 (6.5–11.9)	26.1 (23.5–28.7)	13.0 (11.0–15.0)	0.2 (0–0.5)	3.4 (2.3–4.5)
Central region	26.0 (21.9–30.1)	7.1 (4.7–9.5)	28.0 (25.3–30.7)	10.0 (8.2–11.8)	0.1 (0–0.3)	0.2 (0–0.5)
Eastern region	15.9 (12.5–19.3)	0.9 (0.1–1.8)	19.4 (17.1–21.7)	5.2 (3.9–6.5)	0.0	0.0
Western region	12.4 (9.3–15.5)	2.5 (1.0–4.0)	9.6 (7.9–11.3)	7.4 (5.9–9.0)	0.8 (0.2–1.4)	5.9 (4.5–7.3)
High blood cortisol* levels and infected	7.4 (4.9–9.9)	1.2 (0.2–2.2)	21.0 (18.6–23.4)	3.2 (2.2–4.2)	0.4 (0.1–0.8)	0.8 (0.3–1.3)
Moderate blood cortisol* and infected	5.5 (3.4–7.6)	1.4 (0.3–2.5)	9.1 (7.4–10.8)	2.0 (1.2–2.8)	0.0	0.5 (0.1–0.9)
Normal blood cortisol* and infected	26.0 (21.9–30.1)	6.0 (3.8–8.2)	47.7 (44.8–50.7)	10.6 (8.8–12.4)	0.4 (0.1–0.8)	1.9 (1.1–2.7)

CI = Confidence interval. The parasites were recovered from the skin, fins, and/or the gills of infested fish, except for Centrocestus, which was only recovered from the gills.

* Blood cortisol levels for Nile tilapia were ranked as: low cortisol level (<7.6 μg/dL blood), medium cortisol level (7.6 – 10.6 μg/dL), and high cortisol level (>10.6 μg/dL) according to Samaras and Pavlidis [18]. As suggested by Hassan [13], the intensity grading rubric (mild, moderate, or severe) was implemented.

### Parasitology

**Figure 2:**
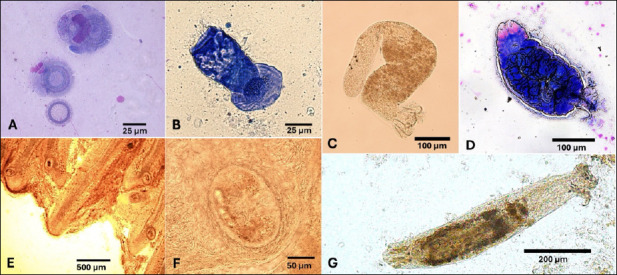
Recovered parasites from naturally infected tilapia sampled from inland farms in Saudi Arabia from 2022 to 2023. A: *Trichodina*, B: *Ambiphyra*, C: *Cichlidogyrus*, D: *Gyrodactylus*, E: *Centrocestus* (multiple cysts), F: *Centrocestus* (single cyst), and G: *Dactylogyrus*. A, B, and D were stained with Giemsa; the rest were not stained.

Parasitic infestations were found on the skin, fins, and gills; no parasites were detected in the eyes or muscles. Six groups of parasites were identified, and at least one group was present in every farm (Table 3 and Figure 2).

Parasite diversity varied:

- One parasite type in one farm (4%) (*Trichodina*)
- Two types in two farms (8%) (*Trichodina* and *Ambiphyra*)
- Three types in 13 farms (52%) (*Trichodina, Cichlidogyrus*, and *Dactylogyrus*)
- Four types in six farms (24%) (*Trichodina*, *Cichlidogyrus*, *Dactylogyrus*, and *Ambiphyra* or *Gyrodactylus*)
- Five types in two farms (8%) (*Trichodina*, *Cichlidogyrus*, *Dactylogyrus*, *Ambiphyra*, and *Gyrodactylus*)

Farms in the central and western regions hosted all six parasite groups. No farms in the eastern region (n = 6) showed *Gyrodactylus* infestations or *Centrocestus metacercariae*. Among all parasites, mild infestations were most common; moderate infestations were less frequent, and severe infestations were rare.

### Key parasite groups

- *Trichodina* spp.: Found on 96% of farms (24/25) and 54.3% of fish. Infestations were mostly mild and more common in winter and in central farms.
- *Cichlidogyrus* spp.: Found on 92% of farms (23/25) and 56.9% of fish, mostly mild infestations.
- *Dactylogyrus* spp.: Found on 80% of farms (20/25) and 22.6% of fish, with a higher prevalence during winter.
- *Gyrodactylus* spp.: Found on 12% of farms, with a low prevalence of 0.9%
- *Centrocestus* spp. (metacercariae): Found on 12% of farms, with mild infestations mainly in central and western regions.
- *Ambiphrya* spp.: Found on nine farms, infecting 10.6% of fish, mostly during winter.

### Predictors of parasitic infections

Logistic regression analysis identified several significant predictors across parasite groups (Table 4). Seasonal effects were evident: summer notably increased the likelihood of infestation with *Centrocestus* (3.6×; p = 0.002), *Ambiphyra* (2.3×; p = 0.048), *Dactylogyrus* (2×; p < 0.001), and *Cichlidogyrus* (1.4×; p = 0.011). Higher temperatures were specifically linked to *Centrocestus* infestation (1.5×; p = 0.001).

**Table 4:** Significant predictors (P) inferred using univariate logistic regression. GLMM of parasite infestations recorded during a one-year cohort study in 25 inland Nile Tilapia aquaculture in Saudi Arabia from 2022 to 2023.

Parasite	Predictor	Univariate Regression					GLMM				

		B	p-value	OR	95% CI		B	p-value	OR	95% CI	
	
					Lower	Upper				Lower	Upper
*Cichlidogyrus*	Season (Summer)	0.368	0.011	1.444	1.092	1.988	0.566	0.270	1.761	–0.441	1.572
	Length (cm)	–0.057	0.026	0.945	0.899	0.993	–0.066	0.150	0.936	–0.155	0.024
	pH	1.705	<0.001	5.501	2.013	15.032	–1.090	0.398	0.336	–3.620	1.440
*Dactylogyrus*	Season (Summer)	0.692	<0.001	1.998	1.547	2.581	1.021	0.009	2.776	0.252	1.790
	pH	1.953	<0.001	7.051	2.761	18.008	–2.534	0.002	0.079	–4.143	–0.925
	DO	–0.120	0.011	0.887	0.808	0.973	0.178	0.028	1.195	0.020	0.337
	Cortisol	–0.025	0.020	0.976	0.956	0.996	0.024	0.995	1.024	–311.747	311.795
*Trichodina*	pH	–4.073	0.007	0.017	0.001	0.328	0.833	0.490	2.301	0.215	24.628
	Cortisol	0.058	0.018	1.060	1.010	1.113	–0.036	0.007	0.965	0.941	0.990
*Centrocestus*	Season (Summer)	1.282	0.002	3.605	1.521	7.652	2.418	0.002	11.229	2.510	50.237
	Length (cm)	–0.165	0.025	0.848	0.723	0.976	0.023	0.861	1.024	0.788	1.329
	Temperature	0.431	<0.001	1.539	1.222	2.004	–0.151	0.359	0.860	0.617	1.199
	DO	0.612	0.006	1.844	1.233	3.114	–0.628	0.049	0.534	0.286	0.995
*Ambiphyra*	Season (Summer)	0.818	0.048	2.266	0.919	5.179	–1.364	0.354	0.256	0.014	4.626
	pH	3.136	<0.001	23.004	2.423	708.014	–0.700	0.797	0.497	0.002	138.098

GLMMGLMM = Generalized Linear Mixed Model, B = Beta coefficient, OR = Odds ratio, 95% CI = 95% confidence interval, DO = Dissolved oxygen.

Low pH strongly predicted infestations with *Ambiphyra* (23×; p < 0.001), *Dactylogyrus* (7×; p < 0.001), and *Cichlidogyrus* (5.5×; p < 0.001), while it was negatively associated with *Trichodina* (0.02×; p = 0.007). DO levels showed positive associations with *Centrocestus* (1.8×; p = 0.006) but negative associations with *Dactylogyrus* (0.9×; p = 0.011).

Fish length was inversely related to *Cichlidogyrus* (p = 0.026) and *Centrocestus* (p = 0.025), indicating that smaller fish were more susceptible.

Cortisol acted as a parasite-specific predictor: it was positively associated with *Trichodina* (p = 0.018) but negatively associated with *Dactylogyrus* (p = 0.02).

GLMMs confirmed:

- Summer as a predictor for *Centrocestus* (11.2×; p = 0.002) and *Dactylogyrus* (2.8×; p = 0.009)
- Low pH was linked with reduced *Dactylogyrus* risk (0.08×; p = 0.002)
- Increased DO with raised *Dactylogyrus* prevalence (1.2×; p = 0.028)
- Increased DO with lower *Centrocestus* prevalence (0.5×; p = 0.049)
- Higher cortisol with decreased *Trichodina* prevalence (p = 0.007)

All parasite-specific GLMMs showed good model fit, as indicated by AIC values.

## DISCUSSION

### Overview of study significance

This study is the first multi-regional and multi-seasonal cohort investigation in Saudi Arabian aquaculture that combines parasitological findings with water quality parameters and physiological stress markers (serum cortisol). Tilapia production in KSA began less than 50 years ago and is expected to remain a key part of national food security. Although disease-free aquaculture systems can theoretically be established, strict biosecurity measures are necessary to prevent the introduction of pathogens [10]. Advanced monitoring programs for viruses, bacteria, and parasites can support infection-free production, but these systems require well-equipped, expensive laboratories that are not always available [18].

### Low-technology parasitology as a biosecurity tool

Given these limitations, the use of low-tech, low-cost screening methods to examine fish or fish eggs before introducing them into new systems is justified. A basic parasitological examination has been recommended as an initial biosecurity measure in inland aquaculture, enabling classification of fish stocks as high-risk batches that should not be added to production systems [18]. Findings from the current study confirm that parasites were found in all examined *O. niloticus* farms, indicating contamination and a risk of pathogen entry. Although not detecting parasites does not guarantee that no viral or bacterial pathogens are present, the presence of parasites clearly indicates a biosecurity breach that needs to be corrected [18]. Therefore, implementing simple, low-cost parasitological screening protocols can significantly enhance national biosecurity surveillance systems.

### Historical introduction and spread of parasites

Geographical variation in parasite occurrence was observed; however, common parasite taxa were widespread throughout the country. This pattern suggests possible dissemination during the early stages of tilapia aquaculture in Saudi Arabia. The first tilapia hatchery, established in Riyadh in 1980 using broodstock imported from Vietnam, began distributing fingerlings to local farms in 1983 [3]. The parasitological status of the original imported stock remains unknown, but these early introductions may have contributed to the subsequent countrywide spread of certain parasite groups in inland aquaculture.

### Potential additional routes of parasite introduction

Although strict national regulations have reduced the chance of importing infested fish, occasional introduction events in previous years cannot be ruled out. Such events could only be confirmed through advanced molecular methods. Parasite spread might also have occurred through the movement of fish between farms within the kingdom. In this study, infestations with *Trichodina* spp. Protozoa were widespread and aligned with previous reports from the eastern [13] and central regions [14, 15, 28]. Other protozoa, such as *Ambiphyra* spp., *Chilodonella* spp., and *Vorticella* spp., were previously documented on central region farms [15, 16, 28]; however, only *Ambiphyra* spp. was observed in this study.

### Role of *Trichodina* as a biosecurity indicator

The ciliated protozoan *Trichodina* has emerged as a promising biological indicator due to its high prevalence, ease of detection, and suitability for rapid, non-lethal screening. Previous research suggested that *Trichodina* loads may reflect the underlying bacterial biomass in water [29], and lowering *Trichodina* levels has been linked to decreasing loads of the bacterial pathogen *Flavobacterium columnare* [30]. In this study, *Trichodina* was found in 96% of the farms surveyed, and it can be detected non-invasively using simple skin smears. Detection does not require advanced equipment; a basic light microscope is enough, and lower-cost field options can be used when needed [31]. Although identifying monogeneans and digeneans requires more detailed taxonomic knowledge, non-invasive methods also enable their detection, supporting their use as practical biosecurity indicators [29].

### Seasonal and regional patterns of parasitic infestations

Previous studies from the central region showed significant variability in the prevalence of monogeneans and *Trichodina* between farms and seasons, ranging from 0% in winter and autumn to 100% in summer [14]. A separate study from the eastern region reported higher *Trichodina* prevalence during cooler seasons [13]. The current study revealed persistent and widespread infestations with *Trichodina* and *Cichlidogyrus* spp. across inland farms, regardless of fish source, water source, season, or location. The long-term persistence of these parasite groups over many years [17] suggests shortcomings in previous biosecurity practices.

### Additional parasitic genera detected and their epidemiological implications

Monogenean infestations have previously been reported in central region tilapia farms [14, 28], and the detection of *Centrocestus* spp. metacercariae in the gills of cultured *O. niloticus* have also been documented [32]. The present study also found encysted metacercariae in the central and western regions but not in the eastern region. The life cycle of *Centrocestus* spp. involves snails as intermediate hosts and birds or mammals as definitive hosts. Historically, *Centrocestus* infections were reported with high prevalence (80%) and heavy intensity in central Saudi Arabia [32], and recent studies in Ethiopia similarly documented high prevalence in *O. niloticus* fingerlings [33]. Snails and potential definitive hosts, including wild birds, dogs, and cats, were observed on the farms in this study. This highlights the need for farm-level biosecurity measures that limit wildlife access to raceways to break the multi-host transmission cycle.

### Dactylogyrus infestations and multispecies farming systems

The detection of *Dactylogyrus* spp., a pathogenic monogenean that favors cyprinid hosts [34, 35], was initially unexpected. However, all farms with *Dactylogyrus* infestations involved multispecies systems stocking common carp (*Cyprinus carpio*), suggesting likely cross-transmission. Stocking density significantly influences stress and growth performance in aquaculture [36], and densely stocked multispecies systems may have facilitated *Dactylogyrus oncomiracidia* settlement on tilapia gills. This discovery highlights the importance of including all farm components, such as species makeup, equipment, and water sources, in biosecurity strategies.

### Parasites, stress response, and environmental interactions

Although multiple parasite species infected cultured *O. niloticus*, infestations were not linked to increased cortisol levels, indicating they did not cause acute physiological stress. However, parasitic effects may worsen when fish encounter environmental stressors, such as poor water quality, higher temperatures, or low DO levels [13]. The absence of a correlation between parasitism and cortisol may also indicate chronic tolerance to ongoing low-level infestations. Further research is necessary to assess potential sampling insensitivity and to identify optimal sampling periods for detecting early stress responses.

### Farm management practices and biosecurity weaknesses

Most farms operated at low to intensive production levels (up to 100 fish/m³), with only 8% using hyper-intensive systems (100–150 fish/m³). Groundwater was the main water source, and farms near natural water bodies might face risks of parasite transfer from naturally infected fish populations [37]. Farms were not connected by a water supply but relied on aeration through propellers and submerged tubes. RAS technology was only active in one farm.

The lack of water filtration, combined with the reuse of untreated effluent from one raceway to another, violates basic biosecurity principles and promotes parasite buildup. Properly assigning nets and tools to individual raceways can help reduce cross-contamination; however, evidence of such practices varied across farms. The current study did not evaluate the impact of chemical treatments on parasite presence.

### Implications for national biosecurity and future directions

The parasite species found in *O. niloticus* in this study belong to commonly reported taxa worldwide [11, 12]. Although genus-level identification was adequate for the study’s goals, molecular confirmation could improve understanding of parasite epidemiology in KSA. The cost-effectiveness of routine parasitological screening outweighs the economic losses from parasitic infections and reduces the need for expensive disease treatments.

An important observation is that several farms did not comply with national biosecurity guidelines [6], including using specific-pathogen-free stocks, following proper quarantine procedures, managing wastewater effectively, using drugs rationally, conducting routine audits, and reporting disease outbreaks. Parasite detection clearly indicates biosecurity breaches, though it does not necessarily identify their root cause, and signals an increased risk of more serious bacterial or viral infections. Comprehensive pathogen screening during all production seasons will require additional sampling and advanced diagnostic methods.

## CONCLUSION

This nationwide, multi-regional, and multi-seasonal assessment of inland *O. niloticus* aquaculture offers the most comprehensive parasitological and environmental baseline currently available for Saudi Arabia. The study found that six parasite groups were present across all surveyed farms, with *Trichodina* spp. and *Cichlidogyrus* spp. being the most common, infecting 54.3% and 56.9% of fish, respectively. Most infestations were mild, but their ongoing presence across regions and seasons suggests a long-standing spread likely originating from early broodstock introductions and maintained through farm-to-farm movements, multispecies systems, and water reuse practices. Key predictors of infestation included season, pH, dissolved oxygen, fish length, and cortisol levels, highlighting the complex interactions among environmental factors, host physiology, and the presence of parasites.

The findings have significant practical implications for national aquaculture biosafety. The widespread presence of easily detectable parasites, especially *Trichodina* spp. and *Cichlidogyrus* spp., supports their use as quick, inexpensive indicators of biosecurity breaches. Since these parasites can be identified through simple, non-lethal wet smears, routine screening can be carried out locally at the farm-level without specialized lab facilities. Incorporating these basic diagnostics into standard procedures can greatly enhance the early warning system of the national biosecurity surveillance program and reduce the risk of introducing fish that may also carry bacterial or viral pathogens.

The study’s main strengths include its multi-seasonal design, extensive geographic coverage, inclusion of water quality parameters and physiological stress markers, and expert-confirmed genus-level parasite identification. These elements offer a realistic and practical view of on-farm health status. However, limitations involve the inability to perform molecular confirmation of parasite species, the omission of virological and bacteriological assessments, and the potential underestimation of stress due to the timing of cortisol sampling. Additionally, the study did not measure environmental host reservoirs, such as snails, or assess the impact of farm-level chemotherapeutants on parasite loads.

Future research should include molecular diagnostics, comprehensive farm biosecurity audits, and long-term pathogen monitoring to better understand transmission pathways. Studies examining the role of intermediate hosts, wildlife interactions, and environmental DNA (eDNA) techniques could help clarify how parasites are introduced and persist. Assessing the cost-effectiveness and adoption rates of on-farm parasite screening programs will support broader implementation at the national level. Additionally, expanding surveillance to include viral, bacterial, and gastrointestinal parasitic pathogens will provide a more complete picture of disease risks in aquaculture.

In conclusion, the widespread and ongoing detection of parasitic infestations across Saudi inland tilapia farms emphasizes the importance of routine, standardized parasitological screening as a key part of biosecurity. While the absence of parasites does not guarantee that stocks are free of other pathogens, their presence clearly indicates a breach that requires immediate corrective action. By adopting scalable, low-cost diagnostic methods, improving farm management practices, and aligning operations with national biosecurity guidelines, the Saudi aquaculture sector can strengthen disease prevention, safeguard production, and promote sustainable growth.

## DATA AVAILABILITY

The supplementary data can be made available from the corresponding author upon request.

## AUTHORS’ CONTRIBUTIONS

MNSA: Writing – review and editing, writing – original draft, software, supervision, resources, project administration, methodology, investigation, formal analysis, conceptualization, and data curation. HIA: Writing – review and editing, methodology, investigation, formal analysis, and data curation. JH and IFA: writing – review and editing, formal analysis, and resources. KB: Writing – review and editing, writing – original draft, methodology, investigation, and formal analysis. All authors have read and approved the final version of the manuscript.
